# Umbilical cord-derived mesenchymal stromal cells: predictive obstetric factors for cell proliferation and chondrogenic differentiation

**DOI:** 10.1186/s13287-017-0609-z

**Published:** 2017-07-05

**Authors:** Léonore Avercenc-Léger, Philippe Guerci, Jean-Marc Virion, Ghislaine Cauchois, Sébastien Hupont, Rachid Rahouadj, Jacques Magdalou, Jean-François Stoltz, Danièle Bensoussan, Céline Huselstein, Loïc Reppel

**Affiliations:** 1 UMR 7365 CNRS-Université de Lorraine, Ingénierie Moléculaire et Physiopathologie Articulaire (IMoPA), Biopôle de l’Université de Lorraine, Campus biologie-santé, Faculté de Médecine, Avenue de la Forêt de Haye, BP 184, 54500 Vandoeuvre-Les-nancy, France; 2 CHRU de Nancy, Unité de Thérapie Cellulaire¸ Banque de Tissus, 54500 Vandœuvre-lès-Nancy, France; 3 Université de Lorraine, 54000 Nancy, France; 4 CHRU de Nancy, Epidémiologie et Evaluation Cliniques, 54500 Vandœuvre-lès-Nancy, France; 5 CHRU de Nancy, Maternité Régionale Universitaire, Département d’Anesthésie-Réanimation, 54000 Nancy, France; 6 UMR 7563 CNRS-Université de Lorraine, LEMTA, 54500 Vandœuvre-lès-Nancy, France; 7 FR3209 CNRS BMCT – Bio-Ingénierie Moléculaire Cellulaire et Thérapeutique, Faculté de Médecine, 54500 Vandœuvre-lès-Nancy, France

**Keywords:** Chondrogenic differentiation, Mesenchymal stromal cells, Obstetric factors, Proliferation, Umbilical cord, Wharton’s jelly

## Abstract

**Background:**

The umbilical cord is becoming a notable alternative to bone marrow (BM) as a source of mesenchymal stromal cells (MSC). Although age-dependent variations in BM-MSC are well described, less data are available for MSC isolated from Wharton’s jelly (WJ-MSC). We initiated a study to identify whether obstetric factors influenced MSC properties. We aimed to evaluate the correlation between a large number of obstetric factors collected during pregnancy and until peripartum (related to the mother, the labor and delivery, and the newborn) with WJ-MSC proliferation and chondrogenic differentiation parameters.

**Methods:**

Correlations were made between 27 obstetric factors and 8 biological indicators including doubling time at passage (P)1 and P2, the percentage of proteoglycans and collagens, and the relative transcriptional expression of Sox-9, aggrecans, and total type 2 collagen (Coll2T).

**Results:**

Amongst the obstetric factors considered, birth weight, the number of amenorrhea weeks, placental weight, normal pregnancy, and the absence of preeclampsia were identified as relevant factors for cell expansion, using multivariate linear regression analysis. Since all the above parameters are related to term, we concluded that WJ-MSC from healthy, full-term infants exhibit greater proliferation capacity. As for chondrogenesis, we also observed that obstetric factors influencing proliferation seemed beneficial, with no negative impact on MSC differentiation.

**Conclusions:**

Awareness of obstetric factors influencing the proliferation and/or differentiation of WJ-MSC will make it possible to define criteria for collecting optimal umbilical cords with the aim of decreasing the variability of WJ-MSC batches produced for clinical use in cell and tissue engineering.

**Electronic supplementary material:**

The online version of this article (doi:10.1186/s13287-017-0609-z) contains supplementary material, which is available to authorized users.

## Background

Due to their capacity for self-renewal, their ability to differentiate into multiple lineages [1], and their immunoregulatory and anti-inflammatory properties [2, 3], mesenchymal stromal cells (MSC) are promising tools for new cell and tissue engineering developments for regenerative medicine and autoimmune/inflammatory disorders.

Bone marrow (BM) is usually regarded as the most common source of adult MSC [4]. MSC isolated from BM (BM-MSC) have been used in up to 200 clinical trials and in various indications (https://www.clinicaltrials.gov; accessed December 2016). However, BM collection is a painful and invasive procedure with the possibility of donor site damage. In addition, it has been demonstrated that the number of available BM-MSC is quite low in this compartment [1], and that in vitro differentiation potential and proliferation capacity decreases with donor age [5–7]. This inter-donor variability is also reported in animal models where the tissue regeneration capacity of MSC isolated from old donors is impaired [8]. Thus, alternative sources of MSC must be considered, including fetal tissue such as the umbilical cord.

Umbilical cord is becoming a notable alternative source of MSC to BM [4]. The conjunctive tissue of the umbilical cord, namely the Wharton’s jelly (WJ), is an abundant and promising source of MSC for clinical applications. Although MSC from Wharton’s jelly (WJ-MSC) share similar characteristics with BM-MSC, they present many advantages: higher frequency and proliferation potential, differentiation in different ways [4, 9–11] and, a priori, no age-dependent variations. Besides, WJ-MSC are more immature according to immunological properties, making them good candidates for allogeneic therapy [12].

The potential for proliferation and in vitro expansion is an essential factor to obtain enough cells to produce clinical batches for patient treatment. Therapeutic doses vary from 1 to 10 × 10^6^ cells/kg, depending on the different clinical protocols (www.clinicaltrials.gov; accessed December 2016). Moreover, it was reported that in vitro culture conditions, such as hypoxia, have a beneficial effect on the proliferative capacities of WJ-MSC [11, 13].

Obstetric factors such as mode of delivery, maternal age, fetal weight, parity, presence of preeclampsia, or hypertension were previously reported as modulating the cells or tissues of the umbilical cord [14–16]. Some studies reported the influence of different obstetric factors on WJ-MSC properties and the main results are summarized in Table 1 [17–23]. This table shows that some obstetric factors such as gestational diabetes could impact not only cell proliferation but also WJ-MSC differentiation potential [18, 19]. In Table 1, several mesodermal and extra-mesodermal differentiations were studied, but none focused on the impact of obstetric factors on chondrogenic differentiation.

**Table 1:** Influence of obstetric factors on Wharton’s jelly-derived mesenchymal stromal cell (WJ-MSC) properties

Obstetric factors	WJ-MSC properties	References
Maternal age	Negative correlation with mesenchymal markers (CD105/CD29) expression	Alrefaei et al. [17]
Gestational diabetes	Improved adipogenic differentiation	Pierdomenico et al. [18]
	Decreased proliferation capacity and viability	Wajid et al. [19]
Obese mothers	Improved adipogenic differentiation	Boyle et al. [20]
Preeclampsia	Improved neuroglial differentiation	Joerger-Messerli et al. [21]
Preterm birth	Similar neuroglial differentiation potential as full-term birth	Messerli et al. [22]
Full-term birth	Enhanced osteoblastic potential	Penolazzi et al. [23]

However, many studies demonstrated the potential for multilineage differentiation of WJ-MSC, especially for chondrogenesis [4, 11, 24–26]. Due to their chondrogenic differentiation potential, WJ-MSC are a promising source of MSC for cell and tissue engineering for cartilage repair and/or regeneration. In three-dimensional cultures, cultivated for 3 to 4 weeks in chondrogenic medium supplemented or not with growth factors (such as transforming growth factor (TGF)-β1 and TGF-β3), several groups including our own reported that WJ-MSC showed chondrogenic induction with expression of specific cartilage-related genes and matrix proteins (Sox9, proteoglycans, type 2 collagen, cartilage oligomeric matrix protein (COMP)) [4, 11, 24–26]. However, a recent study was more reserved, showing poor chondrogenesis from MSC isolated from umbilical cord [27].

As few data on parameters influencing WJ-MSC behavior are available, we wondered whether the donor, i.e., the newborn, and its environment, the mother, and the labor and delivery events influenced WJ-MSC proliferation and differentiation. Whilst the previously mentioned studies described the influence of only one and up to five obstetric factors on cell properties, we sought to evaluate the correlation between a large number of obstetric factors (27), collected during pregnancy and until peripartum, with WJ-MSC proliferation and chondrogenic differentiation parameters. The knowledge of obstetric factors influencing proliferation or differentiation of WJ-MSC will enable us to define the criteria for selecting optimal umbilical cords to be used for the production of clinical batches of WJ-MSC for cell or tissue engineering.

## Methods

### Human umbilical cord harvest and collection of related obstetric factors

Umbilical cords, considered as surgical waste at the time of collection, were obtained after the signing of an informed consent form by pregnant mothers in compliance with French national legislation regarding human sample collection, manipulation, and personal data protection. This collection was approved by the Nancy Hospital ethics committee and French ministry of research (No. DC-2014-2114). Fifty samples were randomly harvested at the Maternity Unit of Nancy University Hospital.

In parallel, for each umbilical cord collected, a large number of related obstetric factors, including maternal-, labor-, delivery-, and newborn-related factors, were recorded anonymously in a database created with FileMaker Pro 11 Advanced software (FileMaker, Santa Clara, CA, USA). After data extraction, only the most relevant and representative obstetric factors, 14 related to the mother, 7 related to the labor and delivery, and 6 related to the newborn, making a total of 27 factors, were used for the study (Additional file 1: Tables S1–S3).

### Isolation and freezing of WJ-MSC

WJ-MSC were isolated as previously described [11]. After collection, umbilical cord samples from fifty donors were rinsed with 70% ethanol and Hanks’ balanced salt solution (HBSS). Umbilical cord vessels were first removed and Wharton’s jelly was aseptically cut into small pieces (2 to 3 mm^3^) plated in a six-well plate with complete medium (minimal essential medium alpha (α-MEM; Lonza, Walkersville, MD, USA) containing 10% fetal bovine serum (FBS), 2 mM glutamine, 100 IU/mL penicillin, 100 μg/mL streptomycin, and 2.5 μg/mL amphotericin B). They were incubated at 37 °C under hypoxic conditions in a tri-gas incubator (MCO-18 M, Sanyo) with humidified gas mixtures of composition <5% O_2_, 5% CO_2_, and >95% N_2_. After 7 days of contact with a plastic surface the cells had migrated and, as enough adherent cells were obtained, pieces were removed, the medium replaced, and cultures continued until cell subconfluence (80–90%). Complete culture medium was changed twice a week and, after 2 weeks, WJ-MSC were harvested with 0.25% trypsin- ethylenediaminetetraacetic acid (EDTA; Sigma-Aldrich, St. Louis, MO, USA). Cells were counted and cryopreserved in FBS with 10% v/v dimethylsulfoxide (DMSO; Sigma) at −80 °C over 24 h and then stored at −150 °C. To control the potential role of pre-thawing parameters on proliferation and chondrogenic differentiation of thawed WJ-MSC, the time to confluence at passage (P)0, the number of cells isolated at the end of P0, and time of cryopreservation were recorded for further analyses.

### Thawing and culture of WJ-MSC

WJ-MSC were quickly thawed, washed with complete medium, and counted with trypan blue (Sigma) dye exclusion test. They were seeded at 1000 cells/cm^2^ in complete medium and cultivated at 37 °C under hypoxic conditions, as previously described. After reaching subconfluence (80–90%), WJ-MSC were harvested with 0.25% trypsin-EDTA (Sigma-Aldrich) and grown up to P2.

### Characterization of WJ-MSC

Characterization of WJ-MSC was performed using five samples. Viability was evaluated just after thawing. Phenotypic analysis, clonogenicity assays, and multilineage differentiation were performed at the end of P2.

#### Viability, apoptosis, and necrosis analysis

Apoptosis and necrosis of cells were analyzed after thawing by flow cytometry using the Vybrant/Apoptosis™ kit based on the Annexin V/propidium iodide (PI) staining procedure (Invitrogen, Carlsbad, CA, USA). Cells were suspended in 100 μL of 1× Annexin-liant buffer with 2.5 μL of Annexin V-Alexa 488 and 1 μL of PI (100 μg/mL) for 15 min at room temperature. After incubation, 200 μL of 1× Annexin V buffer was added to each sample. Cells were then analyzed by measuring fluorescence emission at 530 nm and 575 nm, respectively, for Alexa 488 (apoptotic cells) and PI (necrotic cells) with a Gallios flow cytometer (Beckman Coulter, Brea, CA, USA). Negative (unlabeled cells) and positive controls (apoptosis and necrosis) were performed. For all analyses, at least 5000 events were analyzed. Viable cells were Annexin V and PI negative.

#### Phenotypic analysis

Briefly, to perform phenotypic analysis, WJ-MSC were incubated with fluorescein isothiocyanate (FITC) or phycoerythrin (PE) conjugated mouse anti-human antibodies CD34-PE, CD45-FITC, HLA-DR-FITC, CD90-FITC, CD73-PE, CD105-PE, and CD166-PE (Beckman Coulter) for 30 min at room temperature. Negative and isotype (FITC and PE) controls were performed. After immunofluorescence staining, for each sample, 10,000 events were counted by Gallios flow cytometer (Beckman Coulter).

#### Clonogenicity assays

For colony-forming unit fibroblast (CFU-F) assays, WJ-MSC were harvested and seeded in a six-well plate at 10 cells/cm^2^. They were cultured in complete medium for 10 days in hypoxic conditions. Then, they were washed with phosphate-buffered saline (PBS), stained with cristal violet solution (Sigma) and rinsed with water. CFU-F of more than 50 cells were scored and data expressed as total colony number per 100 cells.

#### Multilineage differentiation

The differentiation potential of WJ-MSC was evaluated under normoxic condition (5% CO_2_, 21% O_2_, 37 °C). Osteogenic and adipogenic differentiation potential were assessed according to the manufacturer’s instructions (Differentiation Media BulletKits, Lonza). To induce osteogenesis, WJ-MSC were harvested and seeded at 3.1 × 10^3^ cells/cm^2^ in complete medium in a 12-well plate. After 24 h, the osteogenesis induction medium (Lonza) was added to the adherent cells; the medium was replaced twice a week and differentiation was continued for 21 days. At day 21, calcium mineralization was assessed by coloration with Alizarin Red (Sigma). For adipogenic differentiation, after harvesting, WJ-MSC were seeded at 2.1 × 10^4^ cells/cm^2^ in complete medium in an eight-well Lab-Tek® (Nunc, Rochester, NY, USA). At 100% confluence, three cycles of induction/maintenance were performed. Each cycle consisted of feeding WJ-MSC with supplemented adipogenesis induction medium (Lonza) and culturing for 3 days, followed by 1–3 days of culture in supplemented adipogenic maintenance medium (Lonza). After 3 complete cycles, WJ-MSC were incubated with adipogenic maintenance medium until 21 days and the medium was replaced twice a week. At day 21, fluorescent staining with AdipoRed™ (Lonza) was performed to detect lipid droplets.

### Proliferation of WJ-MSC

The proliferation capacity of WJ-MSC during monolayer expansion was evaluated using all samples. This parameter was determined at P1 and P2 by the doubling time, according to the following formula:

$$ Doubling\  time\ (h)= T \times \frac{ \log 2}{ \log Ch- \log Cs} $$

where

- T = passage time (h);
- C_h_ = harvested cell number;
- and C_s_ = seeded cell number

### Chondrogenic differentiation of WJ-MSC

For the chondrogenic differentiation step, 2.5 × 10^5^ WJ-MSC from all the samples in P3 were centrifuged in a 15-mL tube at 150 g for 5 min to form a pellet. Chondrogenic differentiation was processed by the three-dimensional culture method and a chondro-inductive medium composed of Dulbecco’s modified Eagle’s medium (DMEM) high glucose 4.5 g/L with l-glutamine supplemented with 100 U/mL penicillin, 100 μg/mL streptomycin, 2.5 μg/mL amphotericin-B (Gibco), 10 μg/mL sodium pyruvate, 5 μg/mL ascorbate, 4 μg/mL l-proline, 2 mM l-glutamine, 10 nM dexamethasone (Sigma), insulin-transferrin-selenium (ITS) + Premix 1% v/v (BD Biosciences, Bedford, MA, USA), and 10 ng/mL TGF-β1 (PeproTech, Rocky Hill, NJ, USA). Cells were incubated during 28 days under hypoxia (5% CO_2_, 2% O_2_, 37 °C) and the chondrogenic medium was changed twice a week.

### Pellet measurements

Pellets were considered as ellipsoidal objects considering their irregular shape. Pellets were measured to obtain their height, width, and depth. The volume was then calculated as follows:

$$ Volume\ \left( m{m}^3\right)=\frac{4}{3}\ \pi abc $$

where

- a = height/2;
- b = width/2;
- and c = depth/2

### Histology and quantification of the matrix synthesis

In preparation for imaging, pellets were fixed in paraformaldehyde 4% p/v phosphate buffered saline (Sigma) and embedded in paraffin. Five micrometer slides were cut and stained with Sirius Red (Sigma) or Alcian Blue (Sigma) and Red Kernechtrot (Merck, Darmstadt, Germany) to determine collagen and proteoglycan synthesis, respectively. Each histological section of pellets was observed by transmitted light Macrofluo Z16APOA A LEICA (Leica, Nanterre, France). Acquisition parameters were defined to be constant, i.e., 65% brightness for Red Sirius staining and 61% brightness for Alcian Blue/Red Kernechtrot. Images were obtained with a LEICA DFC310FX color camera. Images were obtained at 1.51 μm side length square pixel size in 1392 × 1040 matrices at × 5 main objective magnification and × 0.75 macro zoom magnification (combined numerical aperture = 0.125).

A semiquantitative study of the distribution of stained descriptors was processed using Image J (National Institutes of Health, Bethesda, MD, USA). A custom-written Image J program was used to measure the percentage area from the whole section of the pellet. In order to reduce image histogram variability both between and within images, we first used contrast-limited adaptive histogram equalization by using the «Auto Image J» function that will automatically optimize brightness and contrast based on an analysis of the image’s histogram. It was necessary to separate the subject from the background of the image to overcome the contribution to the transmitted light. Then, we proceeded to a chromatic segmentation of each histological stain using the hue, saturation, and brightness properties of the images with the «Color Thresholder ImageJ» function. Images are 8-bit encoded. According to our instrumental settings, the Alcian Blue signature corresponds to the filter hue histogram (137–159). The Red Kernechtrot Chroma is the segmentation interval (195–237). Regarding to the Red Sirius, segmentation was based on the saturation component of the image by filtering the channels (250–255) (Additional file 2: Figure S1).

### Real-time polymerase chain reaction

After 28 days of differentiation, pellets were collected and cryopreserved in 0.5 mL QIAzol (Qiagen, Gaithersburg, MD, USA) before treatment. Total RNA was extracted using the RNeasy Plus Microkit (Qiagen GmbH, Hilden, Germany) following the manufacturer’s instructions, except for the cell lysis made with a Teflon pestle and 200 μL chloroform (Carbo Erba Reagents, Rodano, Milan, Italy) for 1 mL QIAzol. Reverse transcription was performed with 300 ng of total RNA and iScript cDNA Synthesis kit (Biorad, Hercules, CA, USA) following the manufacturer’s instructions in a Thermal Cycler (Biorad). Quantitative polymerase chain reaction (PCR) was made as previously described [26] with the following primers: Sox-9 (forward) 5′-GAG CAG ACG CAC ATC TC-3′ and (reverse) 5′-CCT GGG ATT GCC CCG A-3′; Aggrecans (forward) 5′-TCG AGG ACA GCG AGG CC-3′ and (reverse) 5′-TCG AGG GTG TAG CGT GTA GAG A-3′; total type 2 collagen (Coll2T) (forward) 5′-ATG ACA ATC TGG CTC CCA AC-3′ and (reverse) 5′-GAA CCT GCT ATT GCC CTC TG-3′; and as control, RP29 (forward) 5′-AAG ATG GGT CAC CAG CAG CTC TAC TG-3′ and (reverse) 5′-AGA CGC GGC AAG AGC GAG AA-3′, the expression of which was not modified by the hypoxia culture conditions. Values were normalized to expression of RP29 mRNA.

### Design of the study

Eight biological parameters were analyzed: doubling time at P1 and P2, the volume of pellets, the percentage of proteoglycans and collagens, and the relative transcriptional expression of Sox-9, aggrecans, and Coll2T. Experiments were processed on 50 samples. Two samples could not be analyzed by RT-PCR because of the lack of biological material. Correlations between the 27 obstetric factors and the 8 biological indicators were analyzed.

### Statistical analysis

Control of the role of pre-thawing parameters on the eight biological indicators (doubling time at P1 and P2, volume of the pellets, percentage of proteoglycans and collagens, relative transcriptional expression of Sox-9, aggrecans and Coll2T) were assessed with SAS (SAS Institute, Brie Comte Robert, France) with a Pearson test or a Student’s *t* test. Quantitative and qualitative data of the studied obstetric factors are presented as number of events, with mean and standard deviation when applicable. Correlations between the 27 obstetric factors and the 8 biological indicators were analyzed on the 50 samples with SAS (SAS Institute, Brie Comte Robert, France). All samples were analyzed in bivariate regression, for which mean per class are presented for qualitative variables and correlation coefficient for quantitative variables. One-way analysis of variance (ANOVA) was performed in case of equal variances; if not, Kruskal-Wallis test was performed for qualitative variables and correlation test for quantitative variables. For multivariate regression, only the effects significantly associated at threshold 0.15 in bivariate regression were candidates. The stepwise selection method variable was used with significance level for entering effects at 0.1 and a significance level for removing effects at 0.05. Variables that do not appear in the multivariate regression do not meet those criteria. A statistically significant correlation was assumed for *p* ≤ 0.05.

## Results

### Characterization of WJ-MSC

**Fig. 1:**
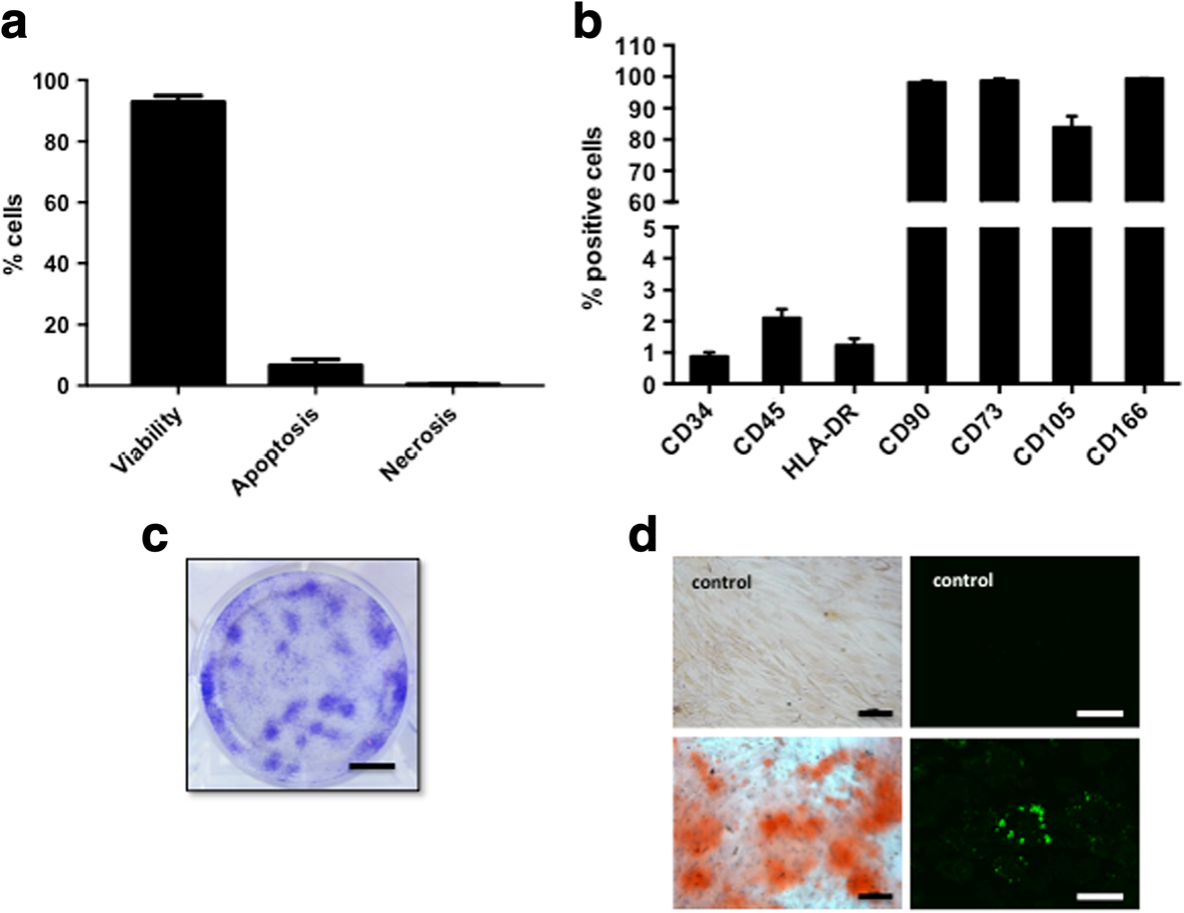
Characterization of WJ-MSC. **a** Viability, apoptosis, and necrosis were evaluated just after thawing. Apoptosis and necrosis of cells was analyzed by flow cytometry using the Vybrant/Apoptosis™ kit based on the AnnexinV/ PI staining procedure. **b** Phenotypic analysis, **c** clonogenicity assays (scale bar = 1 cm), and **d** multilineage differentiation (*scale bar* = 100 μm) were performed at the end of P2. For mesenchymal surface markers, the results are shown as percentages of positive cells. Osteogenic differentiation was evaluated by the matrix calcium mineralization as shown by Alizarin red staining. Adipogenic differentiation was assessed by detection of lipid droplets by a fluorescent staining with AdipoRed™ (*n* = 5)

Cell viability after thawing was higher than 90% (Fig. 1a). Cells adhered to the plastic dishes and had a fibroblastic morphology. They positively expressed mesenchymal markers such as CD73, CD90, CD105, and CD166 with expression levels greater than 80%. The expression of hematopoietic markers CD34 and CD45 was negative, as was that of HLA-DR (Fig. 1b). Clonogenic capacities and mesodermic differentiation potential were confirmed at the end of P2 (Fig. 1c and d).

### Impact of pre-thawing parameters on proliferation and chondrogenic differentiation

Statistical analysis of pre-thawing parameters (time to confluence at P0 and number of cells isolated at the end of P0) showed no impact on biological indicators of proliferation (*p* ≥ 0.2358). Only the time of cryopreservation influenced chondrogenic differentiation through the matrix synthesis of proteoglycans and collagens (*p* ≤ 0.0198) (Additional file 1: Table S4). Thus, this parameter was taken into account in the multivariate analysis of correlations between the 27 obstetric factors and the 8 biological indicators (Table 2).

**Table 2:** Impact of obstetric factors on proliferation and chondrogenic differentiation

Obstetric factors	P1 doubling time (h)	P2 doubling time (h)	Volume (mm^3^)	Proteoglycans (%)	Collagens (%)	Sox-9/ RP29	Aggrecans/RP29	Coll2T/RP29
Birth weight (g)		+						
Amenorrhea weeks at birth		+						
Full-term birth		+						
Maternal smoking		+				–		
Normal pregnancy: neonatal criteria		+						
Preeclampsia		–						
Managed labor	+			+				
Oxytocin infusion	+							
Placental weight		+			–			
Twins				–				
Normal pregnancy (labor and delivery criteria)			+					
Arterial hypertension				+				
Labor duration			–					
Long labor			–			+		
Normal pregnancy (maternal criteria)								+
Cryopreservation duration (days)				+	–			
*R* ^2^ =	0.12	0.14	0.13	0.25	0.25	0.23	0	0.16

+ positive impact (i.e., decreased doubling time or increased marker of chondrogenic differentiation), – negative impact (i.e., increased doubling time of decreased marker of chondrogenic differentiation). *Coll2T* total collagen type 2, *P* passage

### Proliferation

**Fig. 2:**
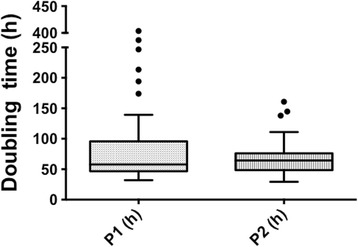
Doubling time. Distribution of sample doubling time at passage 1 (*P1*) and P2. Proliferation capacities of the samples were determined by their doubling time after thawing, at P1 and P2 (*n* = 50)

Cells were seeded at 1000 cells/cm^2^ and incubated until subconfluence (80–90%) was reached. Days of culture were counted and doubling time was calculated in order to analyze cell proliferation potential. Doubling time was reduced between passage 1 and 2 (86.8 ± 75 h vs 68.1 ± 27.4 h). Although the difference was not significant, a marked decrease in the variations between samples was observed (Fig. 2).

### Impact of obstetric factors

**Fig. 3:**
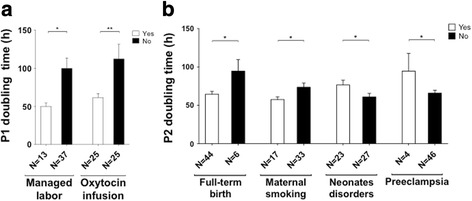
Relationship between obstetric factors and doubling time. **a** Doubling time was calculated at passage 1 (*P1*). Bivariate regression showed an impact of managed labor, which decreased P1 doubling time. Multivariate regression showed an impact of oxytocin infusion, decreasing doubling time. Occurrence of these two obstetric factors during labor is positive for cell proliferation. **b** Doubling time was calculated at P2. Bivariate regression showed a positive impact of full-term birth and maternal smoking and a negative impact of neonate disorders and preeclampsia. *p ≤ 0.05 after bivariate regression analysis; **p ≤ 0.05 after multivariate regression analysis

P1 doubling time was influenced by managed labor (defined by oxytocin infusion and/or artificial rupture of membranes) after bivariate regression analysis (50.0 ± 4.6 h vs 99.8 ± 13.7 h, *p* = 0.0383) and oxytocin infusion after multivariate regression analysis (61.6 ± 5.2 h vs 112.0 ± 19.5 h, *p* = 0.0159), as shown in Fig. 3a. Both significantly decreased P1 doubling time, so their application during labor was positive for cell proliferation. However, none of them impacted P2 doubling time (*p* = 0.3203 and 0.2157 after bivariate regression analysis). Studying factors influencing P1 doubling time according to a threshold of 100 h, several factors made it possible to obtain a P1 less than 100 h such as oxytocin infusion (57.9% vs 25%, *p* = 0.0469), managed labor (34.2% vs 0%, *p* = 0.0185), amenorrhea weeks at birth (39.85 vs 37.92, *p* = 0.0212), maternal smoking (42.1% vs 8.3%, *p* = 0.0313), and placental weight (552.24 vs 481.92, *p* = 0.0446).

For P2 doubling time, birth weight, amenorrhea weeks at birth, full-term birth, maternal smoking, absence of neonate disorders, absence of preeclampsia, and placental weight all had an impact on cell proliferation (*p* ≤ 0.0482 after bivariate regression analysis). As birth weight, amenorrhea weeks, and placental weight increased, P2 doubling time decreased. Similarly, full-term birth, absence of neonate disorders, and maternal smoking were beneficial for proliferation since P2 doubling time also decreased in both cases (respectively 94.5 h vs 64.5 h, 76.5 vs 61 h, and 73.6 h vs 57.5 h; Fig. 3b). However, the onset of preeclampsia increased P2 doubling time (65.8 h vs 94.6 h; Fig. 3b) Amenorrhea weeks at birth was the only factor which influenced P2 doubling time after multivariate regression analysis (*p* = 0.0094).

### Chondrogenic differentiation

**Fig. 4:**
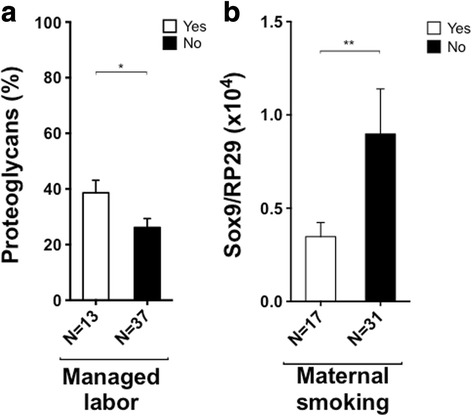
Relationship between obstetric factors impacting P1/P2 and chondrogenic differentiation. **a** Proportion of proteoglycans, stained by Alcian Blue, was measured using a custom-written Image J program. Bivariate regression analysis showed an increase in proteoglycan synthesis by differentiated cells in the presence of managed labor. **b** Sox-9 expression relative to RP29 was assessed by RT-PCR. Multivariate regression analysis showed a significantly reduced expression of Sox-9 at day 28 of differentiation in cells from smoking mothers. *p ≤ 0.05 after bivariate regression analysis; **p ≤ 0.05 after multivariate regression analysis

Obstetric factor effects were first studied on macroscopic biological data after 28 days of chondrogenic induction through pellet volumes, and Alcian blue and Sirius red surface coloration, respectively, for the presence of proteoglycans and collagens. Among obstetric factors that modulated doubling time in P1 and/or P2 only two had an impact on macroscopic criteria for chondrogenic differentiation: managed labor increased the production of proteoglycans in the extracellular matrix (*p* = 0.0440 after bivariate regression analysis; Fig. 4a), and when placental weight increased collagen production decreased (*p* = 0.0230 after bivariate regression analysis). Birth weight, amenorrhea weeks at birth, term birth, no neonate disorders, preeclampsia, and oxytocin infusion did not impact chondrogenic differentiation (*p* ≥ 0.1106). Considering molecular biology parameters, Sox-9 relative expression at day 28 of differentiation was reduced in the event of maternal smoking, which correlated with decreased P2 doubling time (*p* = 0.0367 after multivariate regression analysis; Fig. 4b).

**Fig. 5:**
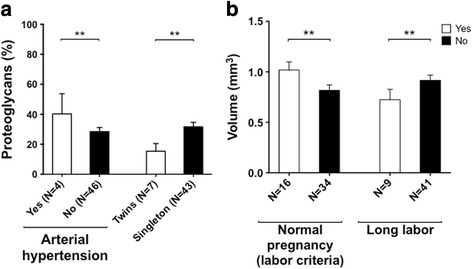
Relationship between obstetric factors nonimpacting P1/P2 and chondrogenic differentiation. **a** Proportion of proteoglycans, stained by Alcian Blue, was measured using a custom-written Image J program. Multivariate regression analysis showed an increase in proteoglycan synthesis by differentiated cells from mothers with arterial hypertension and singletons. **b** Volume of pellets after 28 days of differentiation was measured. Multivariate regression analysis showed an increased volume of pellets in the event of normal pregnancy with regard to labor and delivery criteria. **p ≤ 0.05 after multivariate regression analysis

Chondrogenic differentiation was influenced by six other obstetric factors which did not impact proliferation either positively or negatively (*p* ≤ 0.0447 after bivariate regression analysis or *p* ≤ 0.0480 after multivariate regression analysis; Table 2). Proteoglycan synthesis was increased by arterial hypertension in the mother and decreased by delivery of twins (Fig. 5a). Furthermore, the volume of pellets was increased by normal pregnancy with regard to labor and delivery criteria and decreased by long labor (defined as >8 h for primipara and >6 h for multipara) (Fig. 5b). Relative transcriptional expression of Sox-9 and Coll2T at day 28 of differentiation was increased in the presence of long labor and normal pregnancy. No obstetric factors were found to affect aggrecan mRNA expression.

## Discussion

Given their particular properties, WJ-MSC are promising cells for use in cell and tissue engineering therapy. However, in the context of advanced therapy medicinal product (ATMP) production, criteria which contribute to obtaining reproducible batches of WJ-MSC need to be defined. As donor age is now known to influence BM-MSC expansion, we wondered whether obstetric parameters could also impact WJ-MSC expansion and chondrogenic differentiation. Indeed, some studies previously reported the influence of between one to five obstetric factors on WJ-MSC (Table 1). However, this study is the first to explore the impact of a wide range of obstetric factors (27 obstetric factors related to the mother, the labor and delivery, and the newborn) on WJ-MSC proliferation and chondrogenic differentiation. Due to the variability introduced by cell isolation and cryopreservation, pre-thawing parameters that could affect the biology of thawed cells were taken into account in this analysis of correlations between the 27 obstetric factors and the 8 biological indicators.

In light of planned therapeutic use, cell banking and cryopreservation are necessary steps. Immediate post-thaw use allows MSC to be directly available, abolishing any delay caused by culture expansion. However, cryopreservation remains an inherently stressful process for cells, and could alter cell properties such as viability, phenotype, growth kinetics, differentiation capacities, and immunomodulatory properties. Our study demonstrated that post-thaw viability was higher than 90% and was consistent with recent papers examining the use of cryopreserved MSC [28, 29]. However, we highlighted a long and variable doubling time at P1 (first passage immediately after thawing) compared to P2 (86.8 ± 75 h vs 68.1 ± 27.4 h), suggesting that freeze-thawed cells have impaired proliferative potential. At P1, 23.5% of the samples presented a doubling time higher than 100 h. In contrast, a decreased doubling time was observed at P2, suggesting an improvement in cell proliferative capacity. Restoration of MSC properties after two passages (P2) following thawing was confirmed by cells that exhibited the minimum criteria defining MSC including phenotype, clonogenic, and mesodermic differentiation capacities. The clonogenic potential represents a relevant biological parameter that could also be included in the study of correlation with the obstetric factors. Thus, a culture step seemed useful to restore cell proliferation. However, doubling time at P2 remained significantly higher when P1 was over 100 h (*p* < 0.0084), suggesting that some modifications could not be completely reversed. Different studies reported a controversial impact of cryopreservation on MSC. Several reports demonstrated that cryopreservation did not alter MSC properties in vitro [28, 30, 31] or MSC functionalities in vivo [31, 32] immediately after thawing, whilst other reports claimed that cryopreservation did reduce cell viability [33], proliferative potential [34], and immunomodulatory capacities of MSC [29, 33, 34]. It is therefore relevant to control cell quality after thawing and to define factors that may counteract cryopreservation impairments on cells.

Cell and tissue engineering protocols require large amounts of cells [35], and, as highly proliferative cells, WJ-MSC are a crucial alternative to BM-MSC. Indeed, their collection is painless, non-invasive and more productive, and their immunological features make them the ideal cells for allogeneic therapy. Our study showed that their proliferation could be improved by choosing umbilical cords as a function of certain obstetric factors. Interestingly, proliferation was influenced by different factors at P1 and P2. With regard to cell proliferation, managed labor and oxytocin infusion were the two factors that significantly decreased doubling time at P1. Furthermore, those factors seemed to counteract any impairment related to cryopreservation as doubling time in the oxytocin group was decreased (61.6 ± 5.2 h). Also, we sought a threshold below which certain factors could significantly influence P1. Doubling time at P1 was less than 100 h (57.9% vs 25%, *p* = 0.0469) when parturient women had received an oxytocin infusion. Similarly, we observed that doubling time at P1 was less than 100 h (34.2% vs 0%, *p* = 0.0185) only when managed labor was performed. Oxytocin infusion is related to managed labor as it is used during labor. This neuro-hypophyseal hormone secreted in the hypothalamus with a well-known role in uterine contraction, milk secretion, and cardiovascular functions [36] is the main therapeutic drug frequently used in obstetrics to accelerate labor and birth. Recently, several studies revealed a stimulatory effect of various amounts of oxytocin on the viability and proliferation of undifferentiated and differentiated MSC at P3 [37–39]. Noiseux et al. demonstrated that BM-MSC express the oxytocin receptor, a G-protein-coupled receptor, which activates a mitogen-activated protein kinase, thereby stimulating cell proliferation [39]. In our work, oxytocin administration during labor may have improved cell proliferation directly after thawing. Oxytocin crosses the placental barrier by simple diffusion into the umbilical cord [40] and might therefore interfere with WJ-MSC properties. Oxytocin has a short half-life and its infusion during labor remains punctual leading to only a short impregnation of the umbilical cord. This could explain why the beneficial effect of oxytocin is only observed at P1 and no longer at P2. Other factors made it possible to obtain a P1 less than 100 h such as amenorrhea weeks at birth, maternal smoking, and placental weight. These factors also significantly impact cell proliferation at P2 which clearly indicates that impairment of cryopreservation could hide their effects at P1 in the overall analysis.

The main parameters influencing cell proliferation at P2 were birth weight, amenorrhea weeks at birth, full-term birth, no neonate disorders, placental weight, and absence of preeclampsia. As they are all related to term, with a significant positive impact, our study indicates that full-term birth is a major factor for enhancing cell proliferation of WJ-MSC. Considering the negative impact of preeclampsia on P2 doubling time, a recent study showed the inhibition of human umbilical vein endothelial cell proliferation by early-onset of preeclampsia during pregnancy [41]. This result is consistent with full-term birth since a pregnant mother with preeclampsia rarely carries to term. Accordingly, umbilical cords from parturient women suffering from preeclampsia should not be retained.

Our study showed that maternal smoking had a positive impact on cell proliferation, with regard to P2 doubling time, and a negative impact on Sox-9 expression, considered to be the main transcriptional factor of chondrogenesis, at day 28 of chondrogenic differentiation. Pregnant mothers often underestimate their tobacco consumption because of social pressure, suggesting that tobacco exposure throughout the pregnancy was high. As a component of tobacco, nicotine, which is lipid-soluble and easily diffuses across the placenta [42], might interfere with WJ-MSC properties. Indeed, a recent study showed that prenatal nicotine exposure induces poor quality of articular cartilage [42]. This could be related to the decrease in Sox-9 expression during nicotine exposure and would be consistent with the impaired Sox-9 expression that we observed in smoking mothers. Several studies evaluated the effect of supplementing MSC cultures with various amounts of nicotine on MSC proliferation and chondrogenic differentiation [43–45]. Concerning chondrogenic differentiation from adult MSC, two studies screening different specific markers of chondrogenesis only reported one (type II collagen or aggrecan) increased in the presence of various concentrations of nicotine [43, 44]. Concerning proliferation, Ying et al. showed that nicotine promotes BM-MSC proliferation [43] whereas Zeng et al. reported decreased proliferation of MSC from the umbilical cord in the presence of nicotine in a dose-dependent manner [45]. These contradictory findings may result from experimental conditions as in vivo studies report long nicotine impregnation during pregnancy whereas in vitro studies report only occasional administrations. Unlike oxytocin infusion, nicotine exposure during pregnancy leads to long impregnation that could explain why the impact of maternal smoking is observed at P2. The proliferative effect of maternal smoking during pregnancy has nonetheless to be completed by a study on the functional properties of WJ-MSC.

We assessed chondrogenesis on transcriptional and matrix synthesis steps. Differentiation criteria for the transcriptional step were the expression of Sox-9, a chondrogenesis-specific transcriptional factor, aggrecans, and total type 2 collagen. As for matrix synthesis, the volume of pellets and the presence of proteoglycans/collagens were analyzed. Obstetric factors influencing cell proliferation did not show any significant or global impact, either positive or negative, on chondrogenic differentiation. For example, managed labor that enhanced cell proliferation did have an impact on proteoglycan synthesis but not on any other chondrogenic differentiation criteria. This impact was limited to one criterion of the matrix component and could not lead to a specific conclusion as to the effect of managed labor on chondrogenesis. These results indicate that obstetric factors improving cell proliferation should be considered for cell expansion. Moreover, if the final purpose is cartilage tissue engineering, obstetric factors should be taken into account since they had no negative impact on chondrogenic differentiation. However, other studies showed that MSC differentiation was modulated by the obstetric factors described in our study such as cell proliferation enhancement. Oxytocin inhibited adipogenesis but stimulated osteogenesis, neurogenesis, and angiogenesis of MSC [37, 38, 46, 47]. Moreover, it was reported that full-term birth influences WJ-MSC osteoblastic potential [23] and preeclampsia enhances their neuroglial marker expression [21]. As shown in Table 2, six other obstetric factors influenced one or two criteria of chondrogenesis without influencing cell proliferation. Their effect remained limited and did not impact overall chondrogenesis. In our opinion, many chondrogenesis criteria may be influenced by one or several related obstetric factors.

## Conclusions

In conclusion, we demonstrated a correlation between several obstetric factors and cell proliferation and chondrogenic differentiation parameters. Amongst the obstetric factors considered, multivariate linear regression analysis identified birth weight, the number of amenorrhea weeks, placental weight, normal pregnancy, and absence of preeclampsia as critical factors for cell expansion. All are related to the notion of full-term birth. The WJ-MSC issuing from a healthy term infant showed a greater proliferation capacity. Further investigation could explain the role of managed labor and especially that of oxytocin in cell proliferation and resistance to freezing and, by extension, their importance for cell banking. Regarding chondrogenesis, we showed that obstetric factors acting on proliferation seemed to have a positive effect or no impact on MSC differentiation. It is important to be aware of relevant obstetric factors before harvesting umbilical cord in order to optimize the selection of umbilical cord donors and collect WJ-MSC with most promising properties for use in cell and tissue engineering.

## Additional files

Additional file 1: Table S1. Maternal factors. **Table S2.** Labor and delivery factors. **Table S3** Newborn factors. **Table S4.** Impact of pre-thawing parameters on proliferation and chondrogenic differentiation. (DOCX 47 kb)

Additional file 2: Figure S1. Histological analysis of chondrogenic differentiation. Pellets slides were cut and stained with Alcian Blue and Red Kernechtrot (A–C) or Sirius Red (D–F) to determine proteoglycan and collagen synthesis, respectively. A semiquantitative study of the distribution of stained descriptors was processed using Image J (National Institutes of Health, Bethesda, MD, USA). A custom-written Image J program was used to measure the percentage area from the whole section of the pellet. A chromatic segmentation of each histological stain was performed using the hue, saturation, and brightness properties of the images with the «Color Thresholder ImageJ» function. (DOCX 2164 kb)

## Abbreviations

ATMP: Advanced therapy medicinal product
BM: Bone marrow
CD: Cluster of differentiation
CFU-F: Colony-forming unit fibroblast
Coll2T: Total type 2 collagen
COMP: Cartilage oligomeric matrix protein
DMEM: Dulbecco’s modified Eagle’s medium
DMSO: Dimethylsulfoxide
EDTA: Ethylenediaminetetraacetic acid
FBS: Fetal bovine serum
FITC: Fluorescein isothiocyanate
HBSS: Hank’s balanced salt solution
HLA: Human leukocyte antigen
mRNA: Messenger ribonucleic acid
α-MEM: Minimum essential medium alpha
MSC: Mesenchymal stromal cells
P: Passage
PBS: Phosphate-buffered saline
PE: Phycoerythrin
PI: Propidium iodide
RT-PCR: Real-time polymerase chain reaction
TGF: Transforming growth factor
WJ: Wharton’s jelly
