# Supplementary material for: Umbilical cord-derived mesenchymal stromal cells: predictive obstetric factors for cell proliferation and chondrogenic differentiation
Source: Stem Cell Res Ther. 2017 Jul 5;8:161. doi: 10.1186/s13287-017-0609-z (PMC5497358; doi:10.1186/s13287-017-0609-z)
Supplement: Supplementary file 1 — Maternal factors. Table S2. Labor and delivery factors. Table S3 Newborn factors. Table S4. Impact of pre-thawing parameters on proliferation and chondrogenic differentiation. (DOCX 47 kb) [file 13287_2017_609_MOESM1_ESM.docx]

**Additional file 1**

**Table S1.** Maternal factors

|  | | | | | | | | | | | | | | | | |
| --- | --- | --- | --- | --- | --- | --- | --- | --- | --- | --- | --- | --- | --- | --- | --- | --- |
|  |  | N |  | %/Mean |  | SD ¤ |  | Median |  | Q1 |  | Q3 |  | Min |  | Max |
|  |  |  |  |  |  |  |  |  |  |  |  |  |  |  |  |  |
| ***Age** | | 50 |  | 29.84 |  | 4.40 |  | 30.00 |  | 27.00 |  | 32.00 |  | 20.00 |  | 42.00 |
| **Gravidity** | | | | | | | | | | | | | | | | |
|  | Missing | 1 |  |  |  | | | | | | | | | | | |
|  | 1 | 17 |  | 34.7 |  | | | | | | | | | | | |
|  | 2 | 17 |  | 34.7 |  | | | | | | | | | | | |
|  | 3 | 8 |  | 16.3 |  | | | | | | | | | | | |
|  | 4 | 5 |  | 10.2 |  | | | | | | | | | | | |
|  | 5 | 2 |  | 4.1 |  | | | | | | | | | | | |
| **Primigravida** | | | | | | | | | | | | | | | | |
|  | Missing | 1 |  |  |  | | | | | | | | | | | |
|  | No | 26 |  | 53.1 |  | | | | | | | | | | | |
|  | Yes | 23 |  | 46.9 |  | | | | | | | | | | | |
| **Height (m)** | | 50 |  | 1.64 |  | 0.07 |  | 1.65 |  | 1.60 |  | 1.69 |  | 1.51 |  | 1.78 |
| ***BMI (kg/m²)** | | 50 |  | 25.45 |  | 7.00 |  | 23.79 |  | 20.43 |  | 30.09 |  | 17.16 |  | 45.91 |
| **BMI categories** | | | | | | | | | | | | | | | | |
|  | Underweight | 5 |  | 10.0 |  | | | | | | | | | | | |
|  | Normal range | 25 |  | 50.0 |  | | | | | | | | | | | |
|  | Pre-obese | 7 |  | 14.0 |  | | | | | | | | | | | |
|  | Obese class I | 6 |  | 12.0 |  | | | | | | | | | | | |
|  | Obese class II | 5 |  | 10.0 |  | | | | | | | | | | | |
|  | Obese class III | 2 |  | 4.0 |  | | | | | | | | | | | |
| **Weight (kg)** | | 50 |  | 81.34 |  | 17.43 |  | 77.00 |  | 70.00 |  | 95.00 |  | 49.00 |  | 119.00 |
| **Weight before pregnancy (kg)** | | 50 |  | 68.66 |  | 18.79 |  | 61.00 |  | 55.00 |  | 81.00 |  | 39.50 |  | 125.00 |
| **Weight gain (kg)** | | 50 |  | 12.68 |  | 5.05 |  | 13.00 |  | 10.00 |  | 16.00 |  | -6.00 |  | 23.00 |
| ***Maternal smoking** | | | | | | | | | | | | | | | | |
|  | No | 33 |  | 66.0 |  | | | | | | | | | | | |
|  | Yes | 17 |  | 34.0 |  | | | | | | | | | | | |
| ***Asthma** | | | | | | | | | | | | | | | | |
|  | No | 45 |  | 90.0 |  | | | | | | | | | | | |
|  | Yes | 5 |  | 10.0 |  | | | | | | | | | | | |
| ***Singleton / Twins** | | | | | | | | | | | | | | | | |
|  | No | 43 |  | 86.0 |  | | | | | | | | | | | |
|  | Yes | 7 |  | 14.0 |  | | | | | | | | | | | |
| ***Normal pregnancy (all criteria)** | | | | | | | | | | | | | | | | |
|  | No | 46 |  | 92.0 |  | | | | | | | | | | | |
|  | Yes | 4 |  | 8.0 |  | | | | | | | | | | | |
| ***Normal pregnancy (neonatal criteria)** | | | | | | | | | | | | | | | | |
|  | No | 23 |  | 46.0 |  | | | | | | | | | | | |
|  | Yes | 27 |  | 54.0 |  | | | | | | | | | | | |
| ***Normal pregnancy (maternal criteria)** | | | | | | | | | | | | | | | | |
|  | No | 41 |  | 82.0 |  | | | | | | | | | | | |
|  | Yes | 9 |  | 18.0 |  | | | | | | | | | | | |
| ***Normal pregnancy (labor and delivery criteria)** | | | | | | | | | | | | | | | | |
|  | No | 34 |  | 68.0 |  | | | | | | | | | | | |
|  | Yes | 16 |  | 32.0 |  | | | | | | | | | | | |
| ***Arterial hypertension** | | | | | | | | | | | | | | | | |
|  | No | 42 |  | 84.0 |  | | | | | | | | | | | |
|  | Yes | 8 |  | 16.0 |  | | | | | | | | | | | |
| ***Preeclampsia** | | | | | | | | | | | | | | | | |
|  | No | 46 |  | 92.0 |  | | | | | | | | | | | |
|  | Yes | 4 |  | 8.0 |  | | | | | | | | | | | |
| **Preeclampsia occurrence (AW)** | | 4 |  | 33.13 |  | 5.18 |  | 33.30 |  | 28.65 |  | 37.60 |  | 28.30 |  | 37.60 |
| ***Diabetes mellitus** | | | | | | | | | | | | | | | | |
|  | No | 43 |  | 86.0 |  | | | | | | | | | | | |
|  | Yes | 7 |  | 14.0 |  | | | | | | | | | | | |
| **Premature delivery threat** | | | | | | | | | | | | | | | | |
|  | No | 47 |  | 94.0 |  | | | | | | | | | | | |
|  | Yes | 3 |  | 6.0 |  | | | | | | | | | | | |
| **Premature delivery threat occurrence (AW)** | | 3 |  | 29.83 |  | 1.62 |  | 30.10 |  | 28.10 |  | 31.30 |  | 28.10 |  | 31.30 |
| **Anemia** | | | | | | | | | | | | | | | | |
|  | No | 27 |  | 54.0 |  | | | | | | | | | | | |
|  | Yes | 23 |  | 46.0 |  | | | | | | | | | | | |
| ***Dysthyroidism** | | | | | | | | | | | | | | | | |
|  | No | 44 |  | 88.0 |  | | | | | | | | | | | |
|  | Yes | 6 |  | 12.0 |  | | | | | | | | | | | |
| ***Inflammatory disease** | | | | | | | | | | | | | | | | |
|  | No | 46 |  | 92.0 |  | | | | | | | | | | | |
|  | Yes | 4 |  | 8.0 |  | | | | | | | | | | | |
|  | | | | | | | | | | | | | | | | |
|  |  |  |  |  |  |  |  |  |  |  |  |  |  |  |  |  |

¤: standard deviation; *: factors used for the following study; AW: Amenorrhea weeks

**Table S2.** Labor and delivery factors

|  | | | | | | | | | | | | | | | | |
| --- | --- | --- | --- | --- | --- | --- | --- | --- | --- | --- | --- | --- | --- | --- | --- | --- |
|  |  | N |  | %/Moy |  | SD ¤ |  | Median |  | Q1 |  | Q3 |  | Min |  | Max |
|  |  |  |  |  |  |  |  |  |  |  |  |  |  |  |  |  |
| **Birth season** | | | | | | | | | | | | | | | | |
|  | Winter | 25 |  | 50.0 |  | | | | | | | | | | | |
|  | Spring | 25 |  | 50.0 |  | | | | | | | | | | | |
| ***Vaginal delivery** | | | | | | | | | | | | | | | | |
|  | No | 16 |  | 32.0 |  | | | | | | | | | | | |
|  | Yes | 34 |  | 68.0 |  | | | | | | | | | | | |
| ***Induced labor** | | | | | | | | | | | | | | | | |
|  | No | 31 |  | 62.0 |  | | | | | | | | | | | |
|  | Yes | 19 |  | 38.0 |  | | | | | | | | | | | |
| ***Managed labor** | | | | | | | | | | | | | | | | |
|  | No | 37 |  | 74.0 |  | | | | | | | | | | | |
|  | Yes | 13 |  | 26.0 |  | | | | | | | | | | | |
| ***Oxytocin infusion** | | | | | | | | | | | | | | | | |
|  | No | 25 |  | 50.0 |  | | | | | | | | | | | |
|  | Yes | 25 |  | 50.0 |  | | | | | | | | | | | |
| **Cesarean delivery (C-section)** | | | | | | | | | | | | | | | | |
|  | Vaginal delivery | 34 |  | 68.0 |  | | | | | | | | | | | |
|  | Planned C-section | 4 |  | 8.0 |  | | | | | | | | | | | |
|  | Unplanned C-section | 12 |  | 24.0 |  | | | | | | | | | | | |
| **C-section etiology** | | | | | | | | | | | | | | | | |
|  | Fetal electrocardiogram alteration | 4 |  | 25.0 |  | | | | | | | | | | | |
|  | Preeclampsia/eclampsia | 2 |  | 12.5 |  | | | | | | | | | | | |
|  | Breech/Transverse lie | 1 |  | 6.3 |  | | | | | | | | | | | |
|  | Arrest of dilatation | 6 |  | 37.5 |  | | | | | | | | | | | |
|  | Post term pregnancy | 1 |  | 6.3 |  | | | | | | | | | | | |
|  | Scarred uterus | 2 |  | 12.5 |  | | | | | | | | | | | |
| ***Labor duration (h)** | | 44 |  | 6.07 |  | 2.87 |  | 5.50 |  | 4.00 |  | 7.75 |  | 1.00 |  | 14.00 |
| ***Long labor** | | | | | | | | | | | | | | | | |
|  | Missing | 6 |  |  |  | | | | | | | | | | | |
|  | No | 35 |  | 79.5 |  | | | | | | | | | | | |
|  | Yes | 9 |  | 20.5 |  | | | | | | | | | | | |
| **Waters breaking duration (h)** | | 45 |  | 7.76 |  | 12.22 |  | 3.00 |  | 1.00 |  | 11.00 |  | 0.00 |  | 48.00 |
| **Antibiotic during labor** | | | | | | | | | | | | | | | | |
|  | No | 27 |  | 54.0 |  | | | | | | | | | | | |
|  | Yes | 23 |  | 46.0 |  | | | | | | | | | | | |
| ***Placental weight (g)** | | 49 |  | 535.02 |  | 106.00 |  | 530.00 |  | 460.00 |  | 600.00 |  | 243.00 |  | 721.00 |
|  | | | | | | | | | | | | | | | | |
|  |  |  |  |  |  |  |  |  |  |  |  |  |  |  |  |  |

¤: standard deviation; *: factors used for the following study

`

**Table S3.** Newborn factors

|  | | | | | | | | | | | | | | | | |
| --- | --- | --- | --- | --- | --- | --- | --- | --- | --- | --- | --- | --- | --- | --- | --- | --- |
|  |  | N |  | %/Moy |  | SD ¤ |  | Median |  | Q1 |  | Q3 |  | Min |  | Max |
|  |  |  |  |  |  |  |  |  |  |  |  |  |  |  |  |  |
| ***Sex** | | | | | | | | | | | | | | | | |
|  | Missing | 1 |  |  |  | | | | | | | | | | | |
|  | M | 21 |  | 42.9 |  | | | | | | | | | | | |
|  | F | 28 |  | 57.1 |  | | | | | | | | | | | |
| ***Birth weight (g)** | | 49 |  | 3 161.80 |  | 649.19 |  | 3 230.00 |  | 2 920.00 |  | 3 480.00 |  | 760.00 |  | 4 310.00 |
| ***Amenorrhea weeks at birth** | | 49 |  | 39.38 |  | 2.56 |  | 39.90 |  | 39.00 |  | 40.90 |  | 28.30 |  | 41.70 |
| ***Full-term birth** | | | | | | | | | | | | | | | | |
|  | No | 6 |  | 12.0 |  | | | | | | | | | | | |
|  | Yes | 44 |  | 88.0 |  | | | | | | | | | | | |
| ***Fetal growth restriction** | | | | | | | | | | | | | | | | |
|  | Missing | 1 |  |  |  | | | | | | | | | | | |
|  | No | 39 |  | 79.6 |  | | | | | | | | | | | |
|  | Yes | 10 |  | 20.4 |  | | | | | | | | | | | |
| **pH** | | 45 |  | 7.24 |  | 0.07 |  | 7.26 |  | 7.20 |  | 7.29 |  | 7.08 |  | 7.36 |
| **Lactates (mmol/L)** | | 44 |  | 3.05 |  | 1.51 |  | 2.45 |  | 2.10 |  | 3.60 |  | 1.50 |  | 7.80 |
| ***Fetal distress in labor** | | | | | | | | | | | | | | | | |
|  | No | 39 |  | 78.0 |  | | | | | | | | | | | |
|  | Yes | 11 |  | 22.0 |  | | | | | | | | | | | |
| **Head circumference (cm)** | | 44 |  | 34.57 |  | 1.16 |  | 35.00 |  | 34.00 |  | 35.00 |  | 31.00 |  | 37.00 |
| **Height (cm)** | | 44 |  | 48.61 |  | 1.99 |  | 49.00 |  | 47.00 |  | 50.50 |  | 44.50 |  | 52.00 |
|  | | | | | | | | | | | | | | | | |
|  |  |  |  |  |  |  |  |  |  |  |  |  |  |  |  |  |

¤: standard deviation ; *: factors used for the following study

**Table S4.** Impact of pre-thawing parameters on proliferation and chondrogenic differentiation

| \| **Time to confluence at P0** \| \| \| \| \| \| \| \| \| \| --- \| --- \| --- \| --- \| --- \| --- \| --- \| --- \| --- \| \|  \|  \| **P0 duration (days)** \| \| \| \| \| \| \| \| Bivariate regression \| \| \|  \| \| Mean/r* \|  \| P** \| \|  \|  \| \| **Doubling time P1 (h)** \|  \| -0,17 \|  \| **0,2431** \|  \| \| **Doubling time P2 (h)** \|  \| -0,17 \|  \| **0,2358** \|  \| \| **Volume (mm³)** \|  \| -0,19 \|  \| **0,1959** \|  \| \| **SOX9/RP29 (x10000)** \|  \| -0,13 \|  \| **0,3670** \|  \| \| **AGGR/RP29 (x10000)** \|  \| -0,16 \|  \| **0,2680** \|  \| \| **COLL2T/RP29 (x10000)** \|  \| -0,11 \|  \| **0,4502** \|  \| \| **Proteoglycans (%)** \|  \| -0,02 \|  \| **0,9057** \|  \| \| **Collagens (%)** \|  \| -0,06 \|  \| **0,6843** \|  \|  \|  \|  \| \|  \|  \|  \|  \|  \|  \|  \|  \|  \|   * Mean per class for qualitative variables, correlation coefficient for quantitative variables.  **One-way ANOVA analysis (equal variances). Kruskal-Wallis test for qualitative variables otherwise. Correlation test for quantitative variables.  **Number of cells isolated at the end of P0** | | | | | | | | |
| --- | --- | --- | --- | --- | --- | --- | --- | --- | --- | --- | --- | --- | --- | --- | --- | --- | --- | --- | --- | --- | --- | --- | --- | --- | --- | --- | --- | --- | --- | --- | --- | --- | --- | --- | --- | --- | --- | --- | --- | --- | --- | --- | --- | --- | --- | --- | --- | --- | --- | --- | --- | --- | --- | --- | --- | --- | --- | --- | --- | --- | --- | --- | --- | --- | --- | --- | --- | --- | --- | --- | --- | --- | --- | --- | --- | --- | --- | --- | --- | --- | --- | --- | --- | --- | --- | --- | --- | --- | --- | --- | --- | --- | --- | --- | --- |
|  |  | **Cells at the end of P0 (x1000)** | | | | | | |
|  |  | Bivariate regression | | |  |  |  |  |
|  |  | Mean/r* |  | P** |  |  |  |  |
|  |  |  |  |  |  |  |  |  |
| **Doubling time P1 (h)** |  | 0,12 |  | **0,4532** |  |  |  |  |
| **Doubling time P2 (h)** |  | -0,05 |  | **0,7312** |  |  |  |  |
| **Volume (mm³)** |  | 0,13 |  | **0,4069** |  |  |  |  |
| **SOX9/RP29 (x10000)** |  | -0,14 |  | **0,3631** |  |  |  |  |
| **AGGR/RP29 (x10000)** |  | -0,14 |  | **0,3631** |  |  |  |  |
| **COLL2T/RP29 (x10000)** |  | 0,04 |  | **0,7901** |  |  |  |  |
| **Proteoglycans (%)** |  | -0,11 |  | **0,4927** |  |  |  |  |
| **Collagens (%)** |  | 0,13 |  | **0,3949** |  |  |  |  |
|  |  |  |  |  |  |  |  |  |

* Mean per class for qualitative variables, correlation coefficient for quantitative variables.

**One-way ANOVA analysis (equal variances). Kruskal-Wallis test for qualitative variables otherwise. Correlation test for quantitative variables.

| **Time of cryopreservation** | | | | | | | | | | | | | | | | | | | | | |
| --- | --- | --- | --- | --- | --- | --- | --- | --- | --- | --- | --- | --- | --- | --- | --- | --- | --- | --- | --- | --- | --- |
|  |  |  | < 800 days | | | | |  | ≥ 800 days | | | | |  |  | |  |  |  |  |  |
|  |  |  | N=32  (64,0%) | | | | |  | N=18  (36,0%) | | | | |  |  |  |  |  |  |  |  |
|  |  |  | N |  | %/Mean |  | SD* |  | N |  | %/Mean |  | SD* |  | P** | |  |  |  |  |  |
|  |  |  |  |  |  |  |  |  |  |  |  |  |  |  |  | |  |  |  |  |  |
| **Doubling time P1 (h)** | |  | 32 |  | 88,31 |  | 73,99 |  | 18 |  | 84,20 |  | 78,97 |  | **0,8548** | |  |  |  |  |  |
| **Doubling time P2 (h)** | |  | 32 |  | 70,87 |  | 31,23 |  | 18 |  | 63,26 |  | 18,66 |  | **0,3510** | |  |  |  |  |  |
| **Volume (mm³)** | |  | 32 |  | 0,88 |  | 0,34 |  | 18 |  | 0,87 |  | 0,32 |  | **0,9056** | |  |  |  |  |  |
| **SOX9/RP29 (x10000)** | |  | 31 |  | 0,59 |  | 0,97 |  | 17 |  | 0,91 |  | 1,37 |  | **0,3562** | |  |  |  |  |  |
| **AGGR/RP29 (x10000)** | |  | 31 |  | 7,48 |  | 7,23 |  | 17 |  | 11,60 |  | 7,92 |  | **0,0744** | |  |  |  |  |  |
| **COLL2T/RP29 (x10000)** | |  | 31 |  | 7,23 |  | 7,89 |  | 17 |  | 14,27 |  | 18,52 |  | **0,0713** | |  |  |  |  |  |
| **Proteoglycans (%)** | |  | 32 |  | 24,70 |  | 20,28 |  | 18 |  | 37,78 |  | 14,41 |  | **0,0198** | |  |  |  |  |  |
| **Collagens (%)** | |  | 32 |  | 90,28 |  | 7,97 |  | 18 |  | 77,06 |  | 13,74 |  | **<0,0001** | |  |  |  |  |  |
|  | | | | | | | | | | | | | | | | | | | | | |
|  |  |  |  |  |  |  |  |  |  |  |  |  |  |  |  |  | |  |  |  |  |

* Standard deviation

** Student’s t-test
