# Supplementary material for: Umbilical cord-derived mesenchymal stromal cells: predictive obstetric factors for cell proliferation and chondrogenic differentiation
Source: Stem Cell Res Ther. 2017 Jul 5;8:161. doi: 10.1186/s13287-017-0609-z (PMC5497358; doi:10.1186/s13287-017-0609-z)
Supplement: Supplementary file 2 — Histological analysis of chondrogenic differentiation. Pellets slides were cut and stained with Alcian Blue and Red Kernechtrot (A–C) or Sirius Red (D–F) to determine proteoglycan and collagen synthesis, respectively. A semiquantitative study of the distribution of stained descriptors was processed using Image J (National Institutes of Health, Bethesda, MD, USA). A custom-written Image J program was used to measure the percentage area from the whole section of the pellet. A chromatic segmentation of each histological stain was performed using the hue, saturation, and brightness properties of the images with the «Color Thresholder ImageJ» function. (DOCX 2164 kb) [file 13287_2017_609_MOESM2_ESM.docx]

**Figure S1.** Histological analysis of chondrogenic differentiation. Pellets slides were cut and stained with Alcian Blue and Red Kernechtrot **(A, B, C)** or Sirius Red **(D, E, F)** to determine respectively proteoglycan and collagen synthesis. A semi-quantitative study of the distribution of stained descriptors was processed using Image J (National Institutes of Health, Bethesda, MD). A custom-written Image J program was used to measure the percentage area from the whole section of the pellet. A chromatic segmentation of each histological stain was performed using the hue, saturation, and brightness properties of the images with the «Color Thresholder ImageJ» function.
